# Socially Transformative Aesthetics: Real-World Evidence of Positive First Impressions After Pan-Facial Aesthetic Treatments

**DOI:** 10.1093/asjof/ojag061

**Published:** 2026-04-29

**Authors:** Steven Dayan, Julia K Garcia, Nazanin Ashourian, Sara Sangha, Vaishali Patel

## Abstract

**Background:**

Minimally invasive, pan-facial aesthetic treatments rejuvenate and improve facial appearance and may impact social interactions and perceptions.

**Objectives:**

This observational pilot study used video recordings and in-person evaluations to assess social perceptions of participants before and after pan-facial treatment.

**Methods:**

Adults receiving pan-facial injectable treatments (ie, onabotulinumtoxinA [Botox Cosmetic, Allergan Aesthetics, Westport, Ireland], hyaluronic acid fillers [Juvéderm; Allergan Aesthetics, Irvine, California], or both) were invited to participate. Three measures were developed to assess perceptions of attractiveness, approachability, naturalness, trustworthiness, healthiness, and age from nonclinical observer, patient participant, and clinician perspectives: the Pan-Facial Treatment Outcome Survey, the Participant Perception of Appearance, and the Clinician Perception of Appearance.

**Results:**

Twenty-seven participants received treatment and provided video recordings, with 16 videos (*n* = 8 participants) selected for the study; 298 individuals enrolled as nonclinical observers. Compared with baseline assessments, nonclinical observer–reported posttreatment scores were significantly higher for perceptions of attractiveness, approachability, naturalness, healthiness, and perceived age (all *P* values < .05). Posttreatment participant- and clinician-reported scores were significantly higher for the same domains as well as trustworthiness (all *P* values < .05). Compared with baseline, participants and the clinician perceived participants as younger compared to their actual age (3.6 years younger and 2.5 years younger, respectively), and nonclinical observers perceived participants as within 6 months of their actual age after pan-facial treatment.

**Conclusions:**

Pan-facial treatments may positively impact social perceptions and self-assessments of facial appearance. These benefits extend beyond physical appearance, including improving overall social interactions and personal well-being.

**Level of Evidence: 5 (Therapeutic):**

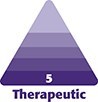

Facial appearance, both during active communication and while at rest, can significantly impact an individual's social interactions, including first impressions and perceptions about their physical health, character traits, and social status.^1,2^ These perceptions affect an individual's self-confidence, psychological well-being, and social functioning, including how they are treated by others.^3,4^ Research has shown that individuals who are considered more attractive and confident are perceived to have more positive character traits and behaviors, such as seeming more approachable or more socially adept, and often experience increased success in the workplace and with interpersonal relationships.^3,5-7^ Improvements in social perceptions can have a far-reaching, meaningful impact, especially on psychosocial well-being—a key motivator for many patients to pursue aesthetic treatments to the face and neck.^3,4^

Pan-facial aesthetic treatments, consisting of 1 or more injectable treatments (eg, neurotoxin and dermal fillers) to the face, are minimally invasive and seek to rejuvenate overall facial appearance and/or improve the shape of the face. These treatments move beyond only targeting specific facial features and instead take a more global approach to treatment.^3,8^ Social perceptions of facial appearance, generally formed by taking into account the entirety of the face (vs individual facial features), are important considerations when evaluating the impact of aesthetic treatments on an individual's self-confidence, psychological well-being, and social functioning. Therefore, observer-reported perceptions are increasingly incorporated into studies to measure treatment benefit. Although much of the evidence demonstrating changes in social perceptions of individuals following aesthetic treatments is focused on surgical treatments, such as orthognathic or facial rejuvenation surgeries,^9-14^ several studies of noninvasive pan-facial aesthetic treatments have found that these noninvasive treatments result in more positive first impressions and social perceptions.^3,15,16^ However, many of these studies have been focused on specific facial areas (eg, forehead lines, glabellar lines) and have not taken into account the entirety of the face.^15-18^ In addition, these studies often use 2-dimensional (2D), static images of participants' faces before and after treatment to assess treatment benefits. Notably, static images alone may not be sufficient to evaluate social perceptions, as alterations to the static face during pan-facial treatment may impact the dynamics and the appearance of the face in motion.

Unlike 2D images, video recordings capture a dynamic face that more closely mirrors real-life, in-person interactions. Using videos to evaluate observer-reported perceptions of participants during active forms of communication, together with clinician-reported perceptions and participants' self-assessments, may provide more a more comprehensive understanding of changes in social perceptions of individuals following aesthetic treatments. Accordingly, this study aimed to assess the impact of pan-facial treatment on social perceptions (specifically, levels of attractiveness, approachability, naturalness, trustworthiness, healthiness, and perceived age) using videos of patient participants before and after treatment as well as participant- and clinician-reported perceptions. We hypothesized that observer assessments of participants' attractiveness, approachability, and healthiness would be higher after receiving pan-facial treatment, and that impressions of trustworthiness and naturalness would stay the same or be higher at posttreatment. Secondary hypotheses were that the clinician providing injections would report increases across all evaluated attributes after administering treatment and that the patient participants would similarly report increases across all attributes at posttreatment.

## METHODS

### Study Design

This observational, single-center, postmarketing pilot study conducted between December 8, 2021, and July 21, 2022, evaluated nonclinical observer–, patient participant–, and clinician–reported perceptions of participants' (ie, patients') faces before and after receiving pan-facial treatments in a real-world clinical practice setting in the United States (US). Adults aged 21 to 65 years who were already planning to receive 1 or more types of minimally invasive, pan-facial injectable treatments were eligible to participate. Participants were not randomized or preassigned to a particular treatment. Treatment options included (1) hyaluronic acid fillers (ie, Juvéderm; Allergan Aesthetics, Irvine, CA) to treat all on-label aesthetic corrections of the midface, jawline, and/or lips; (2) onabotulinumtoxinA (Botox Cosmetic; Allergan Aesthetics, Westport, Ireland) to treat forehead lines, glabellar lines, lateral canthal (crow's feet) lines, and/or masseter hypertrophy; or (3) a combination of both. Participants were excluded if they had received botulinum neurotoxin anywhere on the face within 6 months or any temporary or semipermanent filler injections anywhere to the face or neck within 12 months prior to study entry. In addition to patient participants, for nonclinical observers, adults 18 years of age or older living in the US were recruited via Survey Monkey (SurveyMonkey; San Mateo, CA). The study investigator served as the sole clinician in this pilot study. The participants and observers provided informed consent before completing study activities. The study was approved by Advarra's Institutional Review Board (IRB).

**Figure 1.**
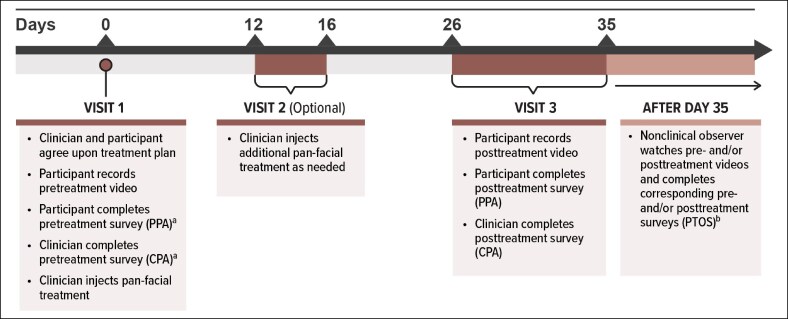
Study design. ^a^Participant- and clinician-reported (participant perception of appearance and clinician perception of appearance) pretreatment surveys were based on in-person evaluations. ^b^Nonclinical observers did not watch pretreatment and posttreatment videos of the same participant. CPA, clinician perception of appearance; PPA, participant perception of appearance; PTOS, pan-facial treatment outcome survey.

Each participant completed 2 to 3 clinical study visits, which is consistent with the number of visits conducted in routine clinical practice for pan-facial treatments. The pan-facial treatment plan most appropriate for each participant was discussed and agreed upon between the clinician and each participant during Visit 1, prior to any discussion related to this research study; the dose, volumes, injection areas, and techniques were at the discretion of the clinician based on the clinician's routine standards of practice. Participants paid out of pocket for their treatment, regardless of the selected treatment. Baseline assessments (including pretreatment participant and clinician measures and pretreatment video recordings) were completed and pan-facial treatment was administered during Visit 1 (Figure 1). Visit 2 was optional, occurring 12 to 16 days after Visit 1, where participants could receive additional treatments at the discretion of the clinician. Final study activities (ie, posttreatment participant and clinician measures and posttreatment video recordings) were completed during Visit 3, which occurred 26 to 35 days after Visit 1. All participant- and clinician-reported measures were completed independently and evaluated the participant's physical and psychosocial status (level of attractiveness, approachability, naturalness, trustworthiness, and healthiness) as well as their perceived age. The same attributes (physical/psychosocial status and perceived age) were evaluated by the nonclinical observers using randomized, anonymous pretreatment and posttreatment video recordings of participants. Each observer independently viewed and rated 8 pretreatment and/or posttreatment videos from the total of 16 videos. Observers never viewed the same participant's pretreatment and posttreatment videos.

### Video Recordings

To ensure standardized video collection procedures, discussion and interview guides were developed, including additional instructions for participants who might have had difficulty performing any of the facial expressions. During all video recordings, participants were asked to demonstrate facial movements associated with happiness, surprise, fear, sadness, and puckering of lips as well as to recite the same nursery rhyme. Videos were then selected for analysis by the study clinician according to the level of consistency in imaging standards (eg, lighting, hair positioning, facial positioning) between a participant's pretreatment and posttreatment videos. Excluded videos were not deemed meaningfully different than the analyzed videos, apart from their reduced imaging quality. All videos were anonymized for use in the nonclinical observer ratings.

### Outcome Measures

The primary outcome measure for this study was the nonclinical observer–reported Pan-Facial Treatment Outcome Survey (PTOS); secondary outcome measures included the participant-reported Participant Perception of Appearance (PPA) questionnaire and the clinician-reported Clinician Perception of Appearance (CPA) questionnaire (Table 1). At the time of this study, there were no published validated observer-reported outcome scales for the assessment of facial aesthetic outcomes; all 3 measures were developed de novo with expert input and have not been formally validated. Each questionnaire contains 6 questions to assess perceptions of participant attractiveness, approachability, naturalness, healthiness, trustworthiness, and age. The PTOS, PPA, and CPA capture responses from the perspective of nonclinical observers (eg, “*How does this person look to you?*”), participants (eg, “*Please look at the overall appearance of your face in the mirror right now…*”), and the clinician (eg, “*Please assess the participant's facial appearance…*”), respectively. Of the 6 questions, 5 use numeric rating scales from 1 to 7, with higher scores indicating more desirable outcomes (1 = *not attractive at all*, *very unapproachable, not natural at all, very unhealthy,* and *very untrustworthy*; 7 = *very attractive*, *very approachable, very natural, very healthy,* and *very trustworthy*). The final question in each measure assessed perceived age; nonclinical observers were asked to provide an estimation of the participant's age (ie, *looks x years old*), and participants and the clinician were asked to rate their perception of the participant's age compared with their actual age (ie, *looks like my/their current age*, *looks older*, or *looks younger*). A negative value indicated a perception that the participant was younger than their current age, and a positive value indicated a perception that the participant was older than their current age.

**Table 1.** Outcome Measures

Survey item	Observer reported (PTOS)	Patient reported (PPA)	Clinician reported (CPA)
Attractiveness^a^	How attractive is this person in the video?	How attractive do you look based on your facial appearance?	How attractive is the participant's facial appearance to you?
Approachability^a^	How approachable does this person look to you?	How approachable do you think you look based on your facial appearance?	How approachable does the participant's facial appearance make them look?
Naturalness^a^	How natural does this person look to you?	How natural do you think you look based on your facial appearance?	How natural does the participant's facial appearance look?
Trustworthiness^a^	How trustworthy does this person look to you?	How trustworthy do you think you look based on your facial appearance?	How trustworthy does the participant's facial appearance make them look?
Healthiness^a^	How healthy does this person look to you?	How healthy do you think you look based on your facial appearance?	How healthy does the participant's facial appearance make them look?
Age^b^	What age does this person appear to be?	How do you think your facial appearance looks compared to your age?	How do you think the participant's facial appearance looks compared to their age?

CPA, clinician perception of appearance; PPA, participant perception of appearance; PTOS, pan-facial treatment outcome survey. ^a^Numeric response scales ranged from 1 to 7, with higher scores indicating more desirable outcomes. Response option 1 = not attractive at all, very unapproachable, not natural at all, very unhealthy, and very untrustworthy. Response option 7 = very attractive, very approachable, very natural, very healthy, and very trustworthy. ^b^Response options included: (1) ____ years (PTOS); (2) I look like my current age, I look ____ years younger, or I look ____ years older (PPA); and (3) The participant looks their current age, the participant looks ____ years younger, or the participant looks ____ years older (CPA).

### Statistical Analyses

All analyses were performed using R, version 4.1.2. Differences in nonclinical observer–reported PTOS scores between groups at pretreatment and posttreatment were calculated using covariate-adjusted linear mixed-effects models and an alpha level of 0.05 for statistical significance. A linear mixed-effects model was fit to each PTOS item, and models were parameterized with each PTOS item as an outcome, with an indicator for pretreatment or posttreatment as a fixed effect. Additionally, differences in participant-reported PPA ratings at pretreatment and posttreatment and clinician-reported CPA ratings at pretreatment and posttreatment were also assessed. Item response distributions were calculated for each PTOS, PPA, and CPA item that used a 7-point numeric rating scale. Distributions were stratified by pretreatment and posttreatment video ratings to compare potential differences following treatment. Inferential tests used case-wise deletion if missingness was present; missing data were not imputed. Perceived age analysis used continuous descriptive methods.

## RESULTS

Participant and observer demographic characteristics are shown in Table 2. Twenty-seven participants received treatment and provided pretreatment and posttreatment videos (see Videos 1 and 2, available online at https://doi.org/10.1093/asjof/ojag061, for pretreatment and posttreatment video examples, respectively). Among these, 16 videos (participants, *n* = 8) were selected by the study clinician for inclusion in the nonclinical observer analysis; video selection was based on the level of consistency in imaging standards between pretreatment and posttreatment videos. Among the 8 participants chosen for the nonclinical observer video analysis, 100% were White and female, their mean age was 35.1 years (standard deviation [SD], 10.3), and their self-reported health status was excellent (*n* = 4, 50%) or very good (*n* = 4, 50%). All 8 participants had a college degree (associate's, 12.5%; bachelor's, 62.5%; master's, 12.5%; doctoral, 12.5%), and 87.5% were working full time. Among participants who received onabotulinumtoxinA, treatment dosage ranged from 6 to 40 units (forehead, 12-40 units; glabellar complex, 15-30 units; masseter muscle, 40 units; lateral canthal lines, 6-12 units per side). Similarly, among participants who received hyaluronic acid fillers, dosage ranged from 0.5 mL to 1.5 mL (cheeks, 0.5-1.5 mL; jawline, 0.5-1.0 mL; lips, 1.0-1.5 mL). A total of 298 individuals enrolled in the study as nonclinical observers. Nonclinical observers were 81.9% White, 51.3% female, and had a mean age of 52.6 years (SD, 17.8). In total, 2384 observer ratings were provided across all participant videos (pretreatment, *n* = 1231; posttreatment, *n* = 1153).

**Table 2.** Participant and Observer Demographic Characteristics

	Patients (*n* = 8)	Nonclinical observers (*n* = 298)
Age,^a^ years		
Mean (SD)	35.1 (10.3)	52.6 (17.8)
Median (IQR)	33.5 (28.8-36.0)	55.0 (37.0-69.0)
Race, *n* (%)		
African American or Black	0 (0.0)	22 (7.4)
American Indian or Alaskan Native	0 (0.0)	4 (1.3)
Asian	0 (0.0)	12 (4.0)
Multiple races	0 (0.0)	11 (3.7)
White	8 (100.0)	244 (81.9)
Hispanic, *n* (%)		
Yes	0 (0.0)	18 (6.0)
No	8 (100.0)	280 (94.0)
Sex, *n* (%)		
Female	8 (100.0)	153 (51.3)
Male	0 (0.0)	139 (46.6)
Other or prefer not to answer	0 (0.0)	6 (2.0)

IQR, interquartile range; SD, standard deviation. ^a^1.7% (*n* = 5) of observers were missing age demographic information.

### Nonclinical Observer-Reported Results: Pan-Facial Treatment Outcome Survey

**Figure 2.**
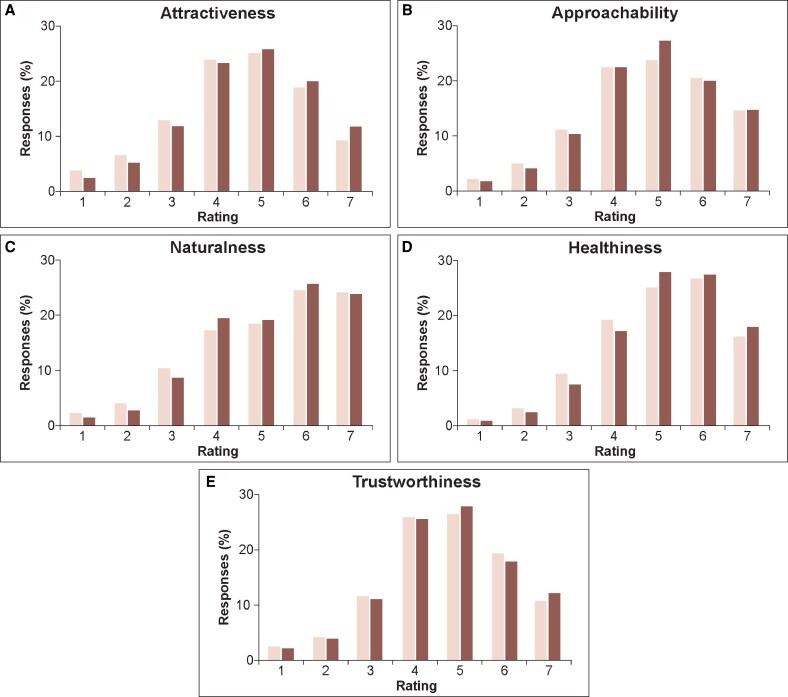
Nonclinical observer–reported (PTOS) results. PTOS, Pan-Facial Treatment Outcome Survey. “Responses (%)” indicates the percentage of respondents that provided a rating of 1-7 for each perception assessed. Rating scores of 1 represented “not attractive at all,” “very unapproachable,” “not natural at all,” “very untrustworthy,” and “very unhealthy.” Rating scores of 7 represented “very attractive,” “very approachable,” “very natural,” “very trustworthy,” and “very healthy.” Response distributions showed a shift toward higher ratings after treatment.

On average, PTOS responses from nonclinical observers for pretreatment and posttreatment videos were concentrated between scores of 4.0 and 7.0 (Table 3; Figure 2). Response distributions showed a shift toward higher ratings after treatment, and no sparsity was observed on any of the 5 PTOS items presented. Compared with baseline, posttreatment PTOS scores were higher and achieved statistical significance for increased attractiveness, approachability, naturalness, healthiness, and perceived age (all *P* values < .05), with an average observer-reported change ranging from 0.09 to 0.20. Specifically, observers rated participants as significantly more attractive (estimated coefficient, 0.20 points; *P* < .001; effect size, 0.138) and approachable (estimated coefficient, 0.09 points; *P* < .001; effect size, 0.062) after treatment, while holding observer-reported age and gender fixed. Similarly, while holding only observer-reported age fixed, observers rated participants with significantly higher healthiness (estimated coefficient, 0.15 points; *P* < .001; effect size, 0.113) and naturalness (estimated coefficient, 0.10 points; *P* = .16; effect size, 0.066). It should be noted that although these differences were statistically significant, the effect sizes were small relative to each item's scale. PTOS posttreatment scores for trustworthiness were slightly higher than baseline scores; however, this finding was not statistically significant (*P* = .082; effect size, 0.051). Standard deviations of the random effects for observers, participants, and the clinician were similar across all items.

**Table 3.** Nonclinical Observer-Reported Results: Pan-Facial Treatment Outcome Survey

	Attractiveness	Approachability	Naturalness	Healthiness	Trustworthiness
Fixed effects, estimate (95% CI)					
Pretreatment (intercept)	3.37 (2.90-3.84)	4.01 (3.58-4.44)	5.15 (4.97-5.32)	4.29 (3.89-4.69)	3.91 (3.50-4.32)
Posttreatment	0.20 (0.12-0.28) *P* < .001 Effect size, 0.138	0.09 (0.01-0.18) *P* = .036 Effect size, 0.062	0.10 (0.02-0.18) *P* = .016 Effect size, 0.066	0.15 (0.07-0.22) *P* < .001 Effect size, 0.113	0.07 (−0.01-0.14) *P* = .082 Effect size, 0.051
Random effects, SD					
Observers	1.11	1.02	1.15	0.97	1.06
Participants	0.23	0.16	0.13	0.21	0.13
Clinician	0.90	1.01	0.97	0.88	0.88

This table shows the linear mixed-effects model assessing the difference between PTOS attractiveness, approachability, naturalness, healthiness, and trustworthiness items between before and after treatment. “Pretreatment” (intercept) is the reference group. Regarding “Posttreatment,” the estimated coefficient for the fixed effect of treatment shows that observers rated the participants’ faces as appearing 0.20 points more attractive after treatment, for example.

CI, confidence interval; SD, standard deviation.

The fixed effect of treatment on perceived participant age showed that observers rated participants within 6 months of their actual current age after treatment (mean, −1.9 months [pretreatment] vs −1.3 months [posttreatment]; estimated coefficient, 0.47 points; *P* = .043; effect size, 0.056), while holding observer-reported age fixed (Table 4). Consistent with the previous PTOS items, the effect size was relatively small.

**Table 4.** Nonclinical Observer, Participant, and Clinician Ratings of Perceived Age

	No. of ratings	Age rating^a^	
		Mean (SD), years	Median (IQR), years
Nonclinical observer reported (PTOS)			
Pretreatment	1231	−1.9 (8.23)	−2.0 (−7.0 to −2.5)
Posttreatment^b^	1153	−1.3 (8.27)	−1.0 (−6.0 to −4.0)
Participant reported (PPA)			
Pretreatment	8	−1.6 (2.33)	0.0 (−3.5 to 0.0)
Posttreatment	8	−3.6 (2.39)	−5.0 (−5.0 to 2.2)
Clinician reported (CPA)			
Pretreatment	8	−0.4 (2.26)	0.0 (−0.5 to 0.5)
Posttreatment	8	−2.5 (3.30)	−0.5 (−5.2 to 0.0)

CPA, clinician perception of appearance; IQR, interquartile range; PPA, participant perception of appearance; PTOS, pan-facial treatment outcome survey; SD, standard deviation. ^a^A negative value indicates a perception that the participant was younger than their current age. A positive value indicates a perception that the participant was older than their current age. ^b^Significance testing was done for the nonclinical observer-reported perceived age of participants (PTOS). The estimated coefficient for the fixed effect of treatment shows that observers rated the participants faces as appearing 0.47 years older after treatment and is statistically significant at *P* = .043 (effect size, 0.056).

### Participant Perception of Appearance

**Figure 3.**
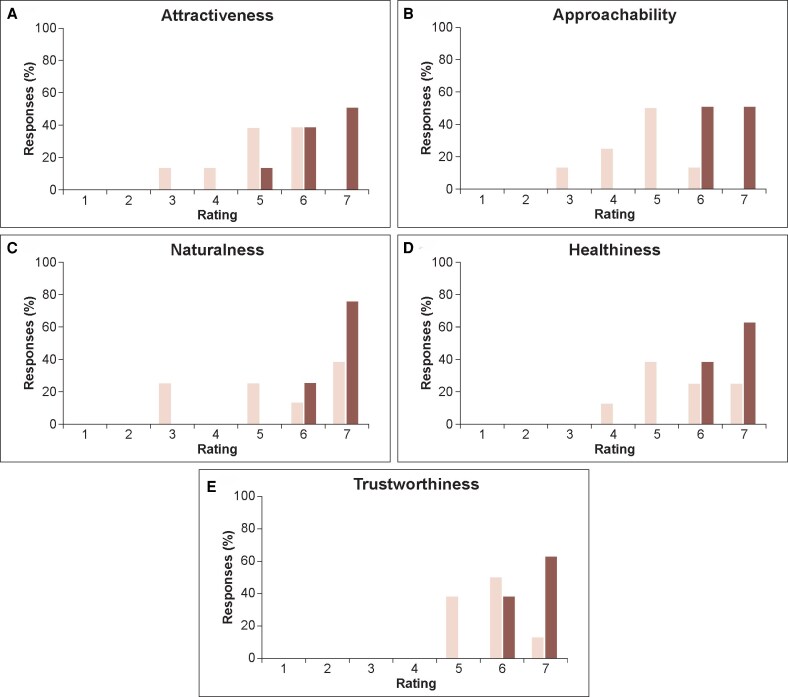
Participant-reported (PPA) results. PPA, participant perception of appearance.

Prior to treatment, participant-reported PPA scores for attractiveness, approachability, and naturalness were concentrated between 3.0 and 6.0. Scores for trustworthiness and healthiness were between 5.0 and 7.0. After treatment, a positive shift was observed in all PPA scores, with participants rating themselves between 5.0 and 7.0 across all items, which resulted in an average self-reported change range of 0.88 to 1.88 (Figure 3). On average, participants rated their perceived age as 1.6 years younger (range, −5.0 to 0.0; SD, 2.3) than their actual age before treatment and 3.6 years younger (range, −6.0 to 0.0; SD, 2.4) after treatment.

### Clinician Perception of Appearance

**Figure 4.**
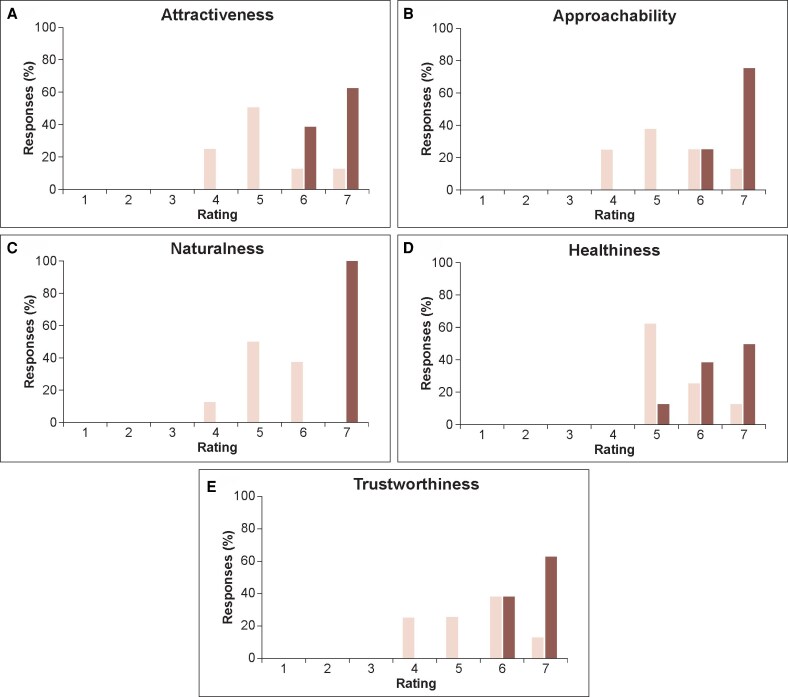
Clinician-reported (CPA) results. CPA, clinician perception of appearance.

Prior to treatment, clinician-reported CPA responses for attractiveness, approachability, naturalness, and healthiness were concentrated between scores of 4.0 and 6.0, whereas scores for trustworthiness were between 5.0 and 7.0. After treatment, a positive shift was observed in all CPA scores, with the clinician rating participants between 6.0 and 7.0 following treatment, which resulted in an average clinician-reported change reported across participants ranging from 0.88 to 1.75 (Figure 4). On average, the clinician rated perceived participant age as 0.4 years younger (range, −5.0 to 2.0; SD, 2.3) than the participant's actual age before treatment and 2.5 years younger (range, −8.0 to 0.0; SD, 3.3) after treatment.

## DISCUSSION

In this real-world pilot study, we found that pan-facial injectable treatments can lead to positive differences in perceptions of attractiveness, approachability, naturalness, trustworthiness, healthiness, and age, as reported by nonclinical observers, participants, and the study clinician. Additionally, these findings show that pan-facial treatments have a favorable impact on social perceptions that are based on physical appearance, emphasizing the importance of considering first impressions when assessing the overall benefit of these treatments.

Recent evaluations of minimally invasive facial aesthetic treatments have begun to use social perceptions as a key consideration to assess treatment effects. In agreement with our findings, these studies reported improved first impressions and observer-reported perceptions at posttreatment.^3,15,16^ However, to date, social perceptions have primarily been assessed via 2D, static images.^15-17^ To our knowledge, this study is the first to utilize video recordings of dynamic faces to examine the impact of minimally invasive pan-facial treatments—for which participants paid out of pocket to reflect real-world clinical practice—by measuring observers' perceptions of participants before and after receiving their pan-facial treatment. This enables a more real-world assessment that incorporates movement and dynamic facial expressions. Using this approach, we found that nonclinical observers' perceptions of participant attractiveness, approachability, naturalness, and healthiness were significantly improved after treatment. Notably, many patients seeking aesthetic treatments desire natural-looking results,^19,20^ and the finding that observers perceived participants as appearing more natural after treatment highlights that the benefits of these treatments extend beyond participants' self-impressions, demonstrating favorable and salient impacts of pan-facial aesthetic treatments on social perceptions.

Although nonclinical observers' perceptions of nearly all of the attributes were significantly improved after treatment, pretreatment and posttreatment observer-reported PTOS scores for trustworthiness did not statistically differ. It may be that trustworthiness is a more complex character attribute that extends beyond evaluations of facial attractiveness, especially when rated alongside other attributes that could be interpreted as more closely related to attractiveness. For instance, Duarte et al^21^ designed a study to evaluate the impact of physical appearance on financial transactions and lending, where observers assumed the role of lender and were asked a series of hypothetical questions based solely on photos of real-life borrowers. They found that, after controlling for physical attractiveness, some faces were still perceived as more trustworthy than others based on first impressions. Further, Olivola and Todorov^22^ identified that face-based social attributions go beyond perceptions of attractiveness through the stereotyping of individuals based on facial features, such as the extent to which an individual has a competent-looking or trustworthy-looking face, regardless of whether they are considered conventionally attractive. For these reasons, pan-facial aesthetic treatments, often intended to improve perceived attractiveness, may have less impact on perceptions of trustworthiness compared with other attributes, particularly for observer-reported evaluations.

With respect to perceived age, the observer-rated change for perceived age was relatively small (6 months), albeit statistically significant. This difference could be due to the open-ended item response format for the observer-reported age question as well as age differences between participants and observers. Notably, although reported identities of race and ethnicity were similar between participants and observers, the mean age of observers was approximately 20 years higher than the mean age of participants. Additional research is warranted to understand the impact of differences in participant and observer demographic characteristics (eg, age, race, sex) on first impressions and social perceptions following aesthetic treatments.

Aligned with previous aesthetics research describing patients' self-evaluations at posttreatment,^23,24^ the results of the participant-reported PPA analyses conducted in this study indicated an increase in positive self-appraisal of psychosocial attributes and perceived age after pan-facial treatment. Similarly, a positive shift was observed in clinician-reported CPA ratings across all items, indicating improved clinician appraisals after treatment and aligning with the pattern observed via PPA analyses. Larger effects were observed in both the PPA and CPA analyses compared with the nonclinical observer-reported PTOS analysis, and there are numerous factors that may explain these differences. For instance, participants may have been primed to report larger differences in their self-perceptions, considering that they sought out and paid out of pocket for treatment. Participants and the clinician may have found it easier to detect more subtle changes in participants' faces, due to familiarity with one's own face as well as clinical expertise and training, compared with nonclinical observers. Additionally, nonclinical observers were the only group blinded to participant treatment, and they could have interpreted measured attributes differently (although instructions were provided to improve consistency across observers). However, it is important to note that these treatments were aesthetic enhancements designed to look natural, and although large differences in social perceptions would not be expected, nonclinical observers' perceptions of nearly all the attributes assessed were significantly improved after treatment.

This study has numerous strengths that should be considered alongside its limitations. Ratings of social perceptions were provided across 3 different perspectives (clinician, participant, and nonclinical observer) to better understand how minimally invasive pan-facial aesthetic treatments impact appearance and social perceptions. In addition, this study was designed to reflect real-world social perceptions of participants following pan-facial aesthetic treatments—participants paid out of pocket for their treatments, data collection occurred during clinic visits conducted in routine clinical practice, and observer perceptions were gathered using video recordings rather than static images. Because participants paid out of pocket for their selected treatments, and some treatment doses were below the label dose, treatment plans reflected what participants were willing to pay and may not be designed to achieve optimal aesthetic outcomes. Limitations of this study include that participants were not selected at random, participant demographics were not representative of the general population, the participant (*n* = 8) and clinician (*n* = 1) sample sizes were small and somewhat homogeneous, and the mean age of observers was nearly 20 years greater than the mean age of participants. Additionally, there may be single investigator bias, as the study clinician performed all injections and selected the videos to be included for analysis. Although the real-world study design increases the generalizability of this study, future larger studies are warranted to increase demographic heterogeneity among participants. The development of validated observer-reported scales, using input from nonclinical observers, patients, and clinicians, should be considered for similar studies in the future; this would allow for clinical interpretation of meaningfulness, as the differences demonstrated in this study, although statistically significant, should be interpreted with caution. Additionally, although this pilot study demonstrated the ability of pan-facial aesthetic treatments to improve social perceptions, future research is needed to understand how these treatments may impact other outcomes of interest, such as confidence, social functioning, and overall well-being.

## CONCLUSIONS

This pilot study found that minimally invasive, injectable, pan-facial aesthetic treatments (ie, onabotulinumtoxinA [Botox Cosmetic; Allergan Aesthetics, Westport, Ireland] and hyaluronic acid fillers [Juvéderm; Allergan Aesthetics, Irvine, CA]) provided natural-looking results that are achievable in real-world practice and were highly rated among participants, the clinician, and nonclinical observers. Additionally, the innovative study design that incorporated social perceptions of dynamic faces into the evaluation of aesthetic treatments may inform the optimization of future studies evaluating similar concepts at scale. Ultimately, these findings suggest that the benefits of such treatments extend beyond patients' self-impressions and can have a broad and favorable impact on social perceptions.
